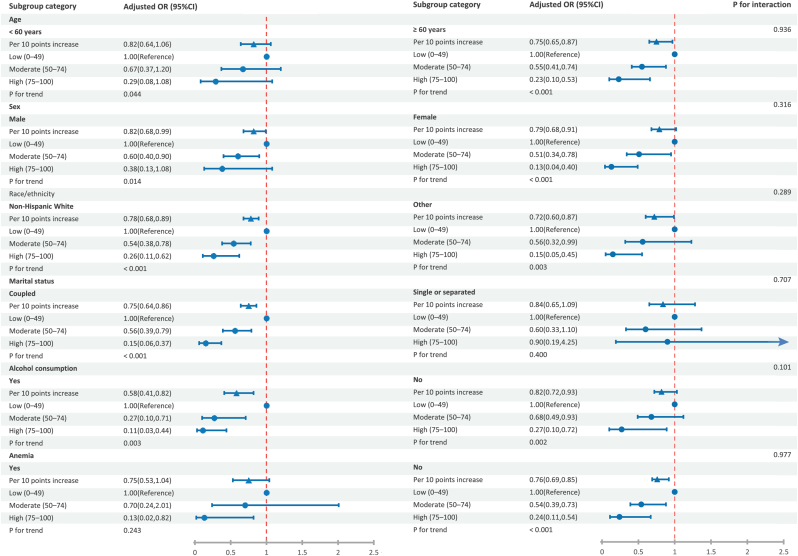# Association between cardiovascular health assessed by Life’s Essential 8 and diabetic retinopathy: The mediating role of phenotypic age and biological age

**DOI:** 10.1016/j.jnha.2025.100711

**Published:** 2025-10-21

**Authors:** Jia Wang, Mingrui Jin, Zhenkang Qiu, Mao Li, Jing Ma

**Affiliations:** a Department of Endocrinology, Gansu Provincial Hospital, Key Laboratory of Endocrine and Metabolic Diseases of Gansu Province, Lanzhou, Gansu, China; b The First School of Clinical Medicine, Lanzhou University, Lanzhou, Gansu, China; c Qingdao Medical College, Qingdao University, Qingdao, Shandong, China; d Beijing Tongren Eye Center, Beijing Tongren Hospital, Capital Medical University, Beijing, China; e Department of Minimally Invasive Interventional Therapy, Sun Yat-sen University Cancer Center, State Key Laboratory of Oncology in South China, Collaborative Innovation Center of Cancer Medicine, Sun Yat-sen University, Guangzhou, Guangdong, China

**Keywords:** Life's essential 8, Diabetic retinopathy, Cardiovascular health, Biological aging markers, NHANES

## Abstract

**Background:**

The American Heart Association (AHA) recently updated the Life's Essential 8 (LE8) metrics to measure ideal cardiovascular health (CVH). Diabetic retinopathy (DR) is the leading cause of vision impairment, exhibiting a rising prevalence globally. However, the association between LE8 and DR, as well as the mediating role of phenotypic age (PA) and biological age (BA), is still unclear.

**Methods:**

LE8 scores were categorized into three groups: low (0–49), moderate (50–74), and high (75–100). PA and BA were computed using validated algorithms incorporating clinical biomarkers. Weighted logistic regression and linear analysis were employed to assess the relationship between LE8 and DR. The mediation analysis was conducted to explore the mediating role of PA and BA.

**Results:**

Our study included 1129 participants with non-DR and 329 participants with DR from the National Health and Nutrition Examination Survey (NHANES). Higher LE8 scores were inversely associated with DR prevalence after adjusting for all covariates (OR = 0.24; 95% CI: 0.11–0.50; *P* for trend < 0.001). Each 10-point LE8 increase was related to a 23% decrease of DR (OR = 0.77, 95% CI: 0.69–0.84). Mediation analysis indicated that PA and BA partially mediated 35.61% and 46.38% of the association between LE8 and DR, respectively.

**Conclusions:**

The LE8 scores were negatively associated with the incidence of DR, while PA and BA partially mediated the association between LE8 scores and DR.

## Highlights

• Higher Life’s Essential 8 (LE8) scores are associated with a lower prevalence of diabetic retinopathy (DR) after covariate adjustment.
• Each 10-point increase in LE8 scores is related to a 23% decrease in the odds of having DR.
• Phenotypic age (PA) and biological age (BA) partially mediate the association between LE8 and DR.

## 1 Introduction

Diabetes mellitus (DM) represents one of the most significant public health challenges worldwide, with approximately 537 million adults aged 20–79 years living with DM in 2021 [1]. This escalating global burden is anticipated to drive a parallel rise in diabetic retinopathy (DR), a common microvascular complication of DM. Epidemiological data reveals that 28.5% of American diabetes patients have DR, with 3.5% suffering vision-threatening DR [2]. Nearly 50% of patients with DR experience functional limitations in at least one visual function task, with deterioration strongly linked to disease progression [3]. These findings emphasize the critical need for DR screening to enable early diagnosis and intervention prior to irreversible visual impairment.

Cardiovascular disease (CVD) remains the leading cause of mortality worldwide and is driven by a set of well-established modifiable risk factors [4]. The American Heart Association (AHA) recently updated the Life's Essential 8 (LE8) metrics to measure ideal cardiovascular health (CVH). It contains smoking, physical activity, obesity, diet, blood lipids, blood pressure, blood glucose, and sleep health metrics [5]. Notably, longitudinal evidence demonstrates that lower LE8 scores exhibited a higher odds of major adverse CVD [6]. Moreover, studies have revealed a robust association between a high CVH score and a reduced risk of developing diabetes [7].

Aging is the deterioration of physiological functions over time, encompassing key regulatory processes such as metabolic homeostasis, inflammatory response, and cellular repair mechanisms, thus accelerating the process of vascular aging [8]. Vascular aging not only serves as a pivotal driver of CVD but also increases the incidence of DR [9]. The occurrence of age-related CVD is influenced by several aging biomarke rs, including telomere length, DNA methylation age, and inflammatory markers [10]. Several common factors contribute to DR, including advanced glycation end products, oxidative stress, and impaired autophagy [11]. Given the intertwined nature of CVH and DR pathogenesis, optimizing CVH represents a promising strategy for mitigating the disease burden of DR. However, the connection between LE8 and DR, as well as the mediating role of phenotypic age (PA) and biological age (BA), is still unclear.

## 2 Materials and methods

### 2.1 Study population and design

The data are derived from the National Health and Nutrition Examination Survey (NHANES) 2005–2008. Data collection methods for the NHANES include in-person interviews, physical examinations, and laboratory tests in two-year cycles. It also uses a complicated multistage probability sampling methodology. All participants provided written informed permission, and the National Center for Health Statistics (NCHS) Ethics Review Board authorized the survey.

### 2.2 Measurement of LE8

Methods for producing the LE8 score were presented in Supplementary Table 1. Each of the eight CVH measures was scored between 0 and 100. LE8 scores were categorized into three groups: low (0–49), moderate (50–74), and high (75–100) [5]. Dietary intake data collected from two 24 -h dietary recalls were combined with United States Department of Agriculture (USDA) food patterns equivalents data to construct and calculate the HEI-2015 scores [12]. The components and scoring standards of Healthy Eating Index (HEI)-2015 are summarized in Supplementary Table 2. Blood samples were collected and analyzed in central laboratories to determine blood lipids, plasma glucose, and hemoglobin A1c levels.

### 2.3 Assessment of DR

Digital retinal pictures of each eye were taken at 45 degrees in individuals. Based on the NHANES Digital Grading Protocol, non-mydriatic fundus photography (TRC-NW6S; Topcon, Tokyo, Japan) was employed in the survey to identify DR. The Early Treatment Diabetic Retinopathy Study (ETDRS) grading scale was used to evaluate the retinal status. Eyes with a higher severity of retinal lesions (Level ≥ 14) were considered to have DR [13].

### 2.4 Ascertainment of biological aging markers

The Klemera-Doubal technique biological age (BA) and Levine method phenotypic age (PA) were two biological aging markers that were estimated using the R package BioAge [14]. The method has been widely validated and applied in many studies [14,15]. In our study, we projected the model to NHANES 2005–2008 data to calculate the corresponding PA and BA. We categorized the biological aging indicators (PA and BA) into tertiles (T1, T2, T3) and used them as independent variables to examine their associations with DR. PA was calculated by using 9 aging-related variables (chronological age, albumin, creatinine, glucose, CRP, lymphocyte percent, mean cell volume, red blood cell distribution width, alkaline phosphatase, and white blood cell count), where xb = −19.907 − 0.0336 × Albumin + 0.0095 × Creatinine + 0.1953 × Glucose + 0.0954 × LnCRP − 0.0120 × Lymphocyte Percent + 0.0268 × Mean Cell Volume + 0.3306 × Red Cell Distribution Width + 0.00188 × Alkaline Phosphatase + 0.0554 × White Blood Cell Count + 0.0804 × Chronological Age.

$$\text{P}\text{h}\text{e}\text{n}\text{o}\text{t}\text{y}\text{p}\text{i}\text{c}\;\text{a}\text{g}\text{e}=\frac{\text{L}\text{n}\left[-0.00553\times \text{L}\text{n}\left(\text{exp}\left(\frac{-1.51714\times \text{exp}\left(\text{x}\text{b}\right)}{0.0076927}\right)\right)\right]}{0.09165}$$

BA was calculated by using 8 biomarkers (Ln-C-reactive protein, serum creatinine, glycosylated hemoglobin, serum albumin, serum total cholesterol, serum urea nitrogen, serum alkaline phosphatase, and systolic blood pressure), where x is the value of biomarker i measured for an individual. For each biomarker i, the parameters k, q, and s are estimated from a regression of chronological age on the biomarker in the reference sample. k, q, and s are the regression intercept, slope, and root mean squared error, respectively.

$$Biological\;age=\frac{\sum_{i=1}^{n}\left(x_{i},-,q_{i}\right)\frac{k_{i}}{s_{i}^{2}}+\frac{CA}{s_{BA}^{2}}}{\sum_{i=1}^{n}{\left(\frac{k_{i}}{s_{i}}\right)}^{2}+\frac{1}{s_{BA}^{2}}}$$

### 2.5 Ascertainment of covariates

Age, gender, race, education level, marital status, alcohol use, calorie intake (kcal/day), and anemia were all included in the statistical model. Studies have demonstrated that anemia is an independent risk factor for the onset and progression of DM [16]. Anemia was defined as hemoglobin levels below 12 g/dL for women and 13 g/dL for men [16].

### 2.6 Statistical analyses

Chi-square tests were performed on categorical variables, and T-tests were conducted on continuous variables. The variance inflation factor (VIF) was used to determine whether multicollinearity existed. When VIF surpassed 10 [17], multicollinearity was considered high. The investigation found no glaring multicollinearity (Supplementary Table 3). Three weighted logistic regression models were built to investigate the connection among the LE8 score, biological aging markers, and DR. Furthermore, weighted logistic regression models also were built to investigate the connection among the LE8 components and DR. A *P* value < 0.05 was used to determine statistical significance for all two-sided statistical tests. R 4.2.2 was used to carry out the analysis.

## 3 Results

### 3.1 Population characteristics

A total of 1458 individuals with 1129 non-DR and 329 DR participants who had diabetes were included in the analyses. The filtering process is shown in Supplementary Fig. 1. Table 1 presents the baseline characteristics of the study population according to the presence or absence of DR. The weighted mean age of 60.52 years and a weighted percentage (WP) of 50.81% for females. Participants with DR were more likely to be male, married, anemic, and less educated, with higher actual age, PA, and BA, lower LE8 scores, blood glucose scores, and blood pressure scores, and higher lipid scores (*P* <  0.05).

**Table 1:** Characteristics of US adults by three categories of diabetic retinopathy. NHANES 2005–2008.^a^

	Overall (*N* = 1458)	Diabetic retinopathy		*P* value
		No (*N* = 329)	Yes (*N* = 1129)	
**Age, years, mean (SE)**	60.52(0.61)	59.75(0.62)	63.75(0.98)	<0.001
**Gender, *n* (%)**				0.032
Female	702(50.81)	548(52.57)	154(43.37)	
Male	756(49.19)	581(47.43)	175(56.63)	
**Race/ethnicity, *n* (%)**				0.022
Non-Hispanic White	749(75.43)	605(77.18)	144(68.06)	
Other	709(24.57)	524(22.82)	185(31.94)	
**Education, *n* (%)**				0.019
Grades 0–12	494(20.96)	360(18.77)	134(30.15)	
High school graduate/GED	365(27.78)	288(28.09)	77(26.49)	
Some colleges or above	599(51.26)	481(53.14)	118(43.36)	
**Marital status, *n* (%)**				0.480
Coupled	891(65.69)	686(65.08)	205(68.25)	
Single or separated	567(34.31)	443(34.92)	124(31.75)	
**Alcohol consumption, *n* (%)**				0.092
Yes	356(25.88)	297(27.23)	59(20.17)	
No	1102(74.12)	832(72.77)	270(79.83)	
**Energy intake, kcal/day, mean (SE)**	1886.08(38.63)	1910.74(43.25)	1782.17(70.58)	0.117
**Anemia**^b^**, *n* (%)**				<0.001
Yes	165(8.56)	102(6.14)	63(18.75)	
No	1293(91.44)	1027(93.86)	266(81.25)	
**Phenotypic age, years, mean (SE)**	56.42(0.68)	54.80(0.68)	63.24(1.10)	<0.001
**Biological age, years, mean (SE)**	46.38(1.09)	43.16(0.88)	59.94(2.44)	<0.001
**Life’s Essential 8 score (out of 100 possible points), mean (SE)**				
Total score	58.75(0.87)	59.68(0.96)	54.82(0.89)	<0.001
Diet score	43.28(1.47)	43.38(1.70)	42.86(2.30)	0.854
Physical activity score	58.41(2.18)	59.67(2.57)	53.09(2.66)	0.077
Nicotine exposure score	69.49(1.72)	69.06(1.79)	71.29(2.29)	0.278
Sleep health score	80.98(1.10)	80.91(1.33)	81.24(1.74)	0.886
Body mass index score	48.23(1.24)	48.54(1.31)	46.95(2.67)	0.574
Blood lipids score	55.82(1.09)	54.79(1.17)	60.16(2.39)	0.046
Blood glucose score	61.79(1.57)	67.01(1.64)	39.77(1.98)	<0.001
Blood pressure score	51.99(1.28)	54.09(1.37)	43.16(2.28)	<0.001

Abbreviations: CVH, cardiovascular health; NHANES, National Health and Nutrition Examination Survey; SE, standard error; GED, general equivalency diploma.

a Means and percentages were adjusted for survey weights of NHANES.

b Anemia was defined as hemoglobin level <13 g/dL in males or <12 g/dL in females.

### 3.2 Associations between LE8 score and DR

The survey-weighted logistic regression models' associations between the LE8 score and the DR are shown in Table 2. In the fully adjusted model (model 2), we found that participants with high LE8 scores had a lower odds of DR than participants with low LE8 scores (OR, 0.24; 95% CI, 0.11–0.50; *P* for trend < 0.001). In the link between LE8 score and DR, the multivariate-adjusted OR for each 10-point increase was 0.77 (95% CI, 0.69–0.84). When the analyses were performed according to the LE8 score's quartile as a sensitivity study, the associations between the LE8 score and DR remained strong (Supplementary Table 4). The restricted cubic spline analyses also revealed that LE8 scores and DR had linear association (*P* for overall < 0.001, *P* for non-linearity > 0.05; Supplementary Fig. 2). Each 10-point LE8 increase was related to a 23% decrease of DR (OR = 0.77, 95% CI: 0.69–0.84).

**Table 2:** Survey-weighted association between Life’s Essential 8 score and diabetic retinopathy.

	Univariable model		Model 1		Model 2	
	OR (95%CI)	P value	OR (95%CI)	P value	OR (95%CI)	P value
Low (0–49)	1[Reference]	/	1[Reference]	/	1[Reference]	/
Moderate (50–74)	0.61(0.48,0.78)	<0.001	0.54(0.43,0.69)	<0.001	0.57(0.44,0.73)	<0.001
High (75–100)	0.22(0.10,0.47)	<0.001	0.21(0.10,0.45)	<0.001	0.24(0.11,0.50)	<0.001
*P* for trend	/	<0.001	/	<0.001	/	<0.001
Per 10 points increase	0.77(0.68,0.88)	<0.001	0.74(0.66,0.83)	<0.001	0.77(0.69,0.84)	<0.001

Model 1 was adjusted for age, gender, race/ethnicity, education level, and marital status.

Model 2 was additionally adjusted for alcohol consumption, energy intake, and anemia.

Abbreviations: OR, odds ratio; CI, confidence interval.

### 3.3 Associations between LE8 components and DR

The relationship between each of the eight CVH metrics and DR was presented in Supplementary Table 5. After adjustment for all covariates, for every ten-score rise in blood glucose scores and blood pressure scores, the multivariate-adjusted ORs of DR were 0.71 (95% CI, 0.65–0.77) and 0.91 (95% CI, 0.86–0.97), respectively.

### 3.4 Associations between LE8 scores and biological aging markers

The survey-weighted linear regression models' associations between LE8 scores and biological age markers are shown in Supplementary Table 6. After controlling for all factors, people with high LE8 scores had younger PA (−6.74; 95% CI, −8.01 to −5.47; *P* for trend < 0.001) and BA (−23.10; 95% CI, −28.67 to −17.52; *P* for trend < 0.001) than people with low LE8 scores. For every ten-score rise in LE8 scores, the multivariate-adjusted Betas of PA and BA reduced by 1.63 (95% CI, −1.93 to −1.33) and 6.10 (95% CI, −7.08 to −5.12), respectively.

### 3.5 Associations between biological aging markers and DR

The associations between biological aging markers and DR were detailed in Supplementary Table 7. Individuals in the T3 of PA and BA were associated with a decreased odds of DR compared to those in the T1 of PA and BA after controlling for all variables (OR, 8.73; 95% CI, 3.41–22.35 and 2.99; 95% CI, 2.04–4.39). Furthermore, the multivariate-adjusted ORs of DR increased by 1.07 (95% CI, 1.05–1.10) and 1.02 (95% CI, 1.01–1.03), with every one-year rise in PA and BA.

### 3.6 Mediation analyses of biological aging markers on associations of LE8 score with DR

**Fig. 1:**
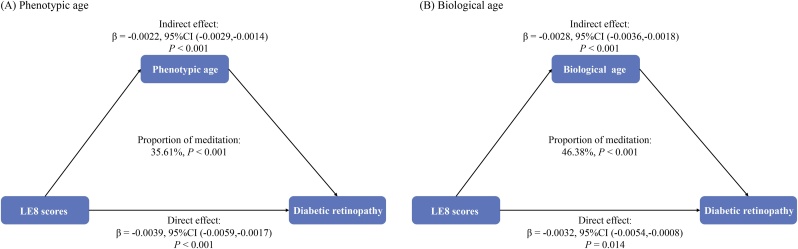
The mediating proportion of biological aging markers on the association between Life’s Essential 8 score and diabetic retinopathy. Adjusted for age, gender, race/ethnicity, education level, marital status, alcohol consumption, energy intake, and anemia. Abbreviations: LE8, Life’s Essential 8; CI, confidence interval.

Parallel mediation analyses were carried out to assess the potential mediation roles of PA and BA on the associations of LE8 score with DR (Fig. 1). Mediation analysis indicated that PA and BA partially mediated 35.61% and 46.38% of the association between LE8 and DR (*P* <  0.001) (Supplementary Table 8).

### 3.7 Subgroup analyses

The subgroup analyses (Supplementary Fig. 3) were conducted to assess whether the association of LE8 score with DR was influenced by age, gender, race, alcohol consumption, and anemia. The stronger inverse associations between categories and continuous LE8 scores and DR were found in older individuals (aged ≥ 60 years), females, other races, married individuals, alcohol users, and anemic patients.

## 4 Discussion

Recent studies observed that as the LE8 score rises, the difference in the odds of experiencing urinary albumin or reduced eGFR decreases significantly [18]. Sun et al. [19] found that LE8 scores are significantly associated with premature mortality, particularly among individuals with T2D. Although the connection between the overall level of CVH and DR has yet to be fully explored, evidence highlights the associations between diet, physical activity, sleep health patterns, obesity, and DR [19,20]. In this study, we found that a higher LE8 score correlates with a lower incidence of DR. Further investigation revealed that PA and BA partially mediate the association.

Several aging biomarkers, including telomere length, DNA methylation, and brain age, were associated with the incidence of T2D [21,22]. Researchers reported a significant association between dysglycemia and retinal age gaps [23]. Additionally, several studies have proved that improving LE8 metrics can effectively mitigate the aging process [24,25]. Tian et al. [26] verified that a higher LE8 score is associated with longer leukocyte telomere length and increased mitochondrial DNA copy number. We hypothesize that improved CVH may be associated with a lower incidence of DR by delaying the aging process. The detailed mechanisms require further prospective studies.

Moreover, we found that not only was an improved blood glucose score significantly associated with a lower incidence of DR, but so was improvement in blood pressure score. Studies have shown that hypertension is an independent risk factor for DR [27]. Furthermore, hypertension status was significantly associated with the severity of DR [28]. Therefore, stringent blood pressure control in diabetes could serve as an preventive strategy to mitigate the odds of experiencing DR. Our research sheds light on the association between LE8 and DR, but it is subject to certain constraints. First, this study was a cross-sectional investigation employing mediation analysis within cross-sectional data. The assessment of eight CVH metrics was limited to baseline measurements, neglecting any alterations that might have taken place throughout the follow-up duration. Additionally, the collection of dietary habits, physical activity levels, nicotine exposure, and sleep health via self-reported questionnaires could lead to recall bias. Extreme caution must be exercised when interpreting the causal implications of our findings. Prospective multicenter studies are needed to corroborate these findings in the future.

## 5 Conclusions

The LE8 scores were negatively associated with the incidence of DR, while PA and BA partially mediated the association between LE8 scores and DR.

## Ethics approval

The survey protocol was approved by the NCHS Ethics Review Board (https://www.cdc.gov/nchs/nhanes/irba98.htm), and all participants have written informed consent.

## Funding

This study was supported by the 10.13039/501100007129Natural Science Foundation of Shandong Province (ZR20230H190), the 10.13039/501100004775Natural Science Foundation of Gansu Province (22JR5RA707), and Project of Gansu Provincial Hospital (24GSSYC-8).

## Declaration of competing interest

**Jia Wang and Ming-rui Jin**: Study Design, Methodology, Statistical Analysis, and Writing of Draft; **Mao Li**: Data Collection, Statistical Supervision and Validation; **Zhen-kang Qiu and Jing Ma**: Conceptualization, Writing-Review, Editing, and Supervision. All authors contributed to the interpretation of the results and critical revision of the manuscript for important intellectual content and approved the final version of the manuscript.

Jing Ma reports financial support was provided by Natural Science Foundation of Gansu Province and Project of Gansu Provincial Hospital. Zhenkang Qiu reports financial support was provided by Natural Science Foundation of Shandong Province. If there are other authors, they declare that they have no known competing financial interests or personal relationships that could have appeared to influence the work reported in this paper

## Figures and Tables

**Figure:**
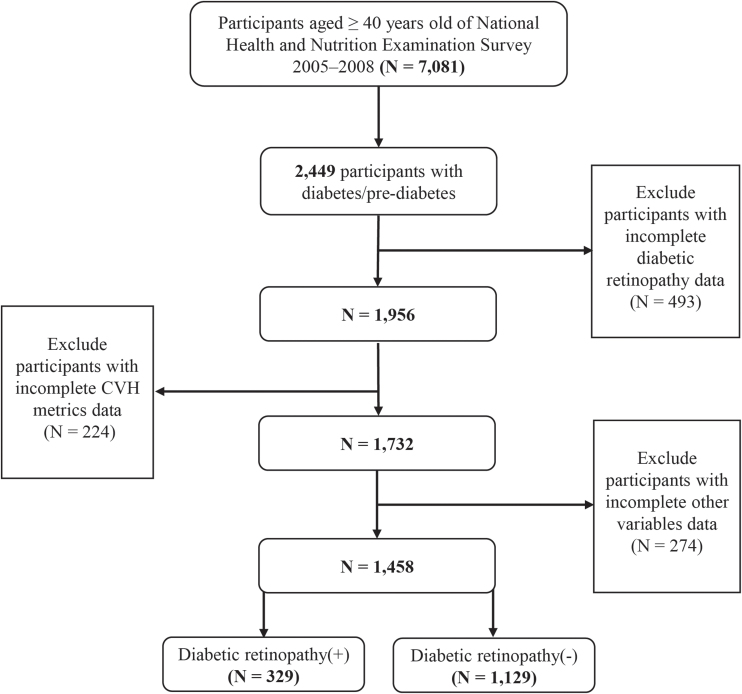


**Figure:**
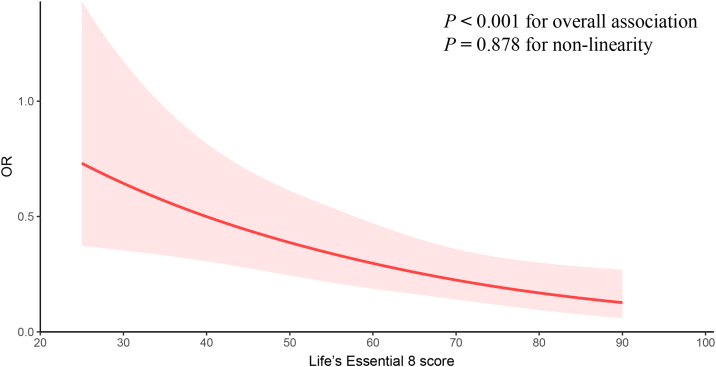


**Figure:**