# Supplementary material for: Association between cardiovascular health assessed by Life’s Essential 8 and diabetic retinopathy: The mediating role of phenotypic age and biological age
Source: J Nutr Health Aging. 2025 Oct 21;29(12):100711. doi: 10.1016/j.jnha.2025.100711 (PMC12642112; doi:10.1016/j.jnha.2025.100711)
Supplement: Supplementary file 1 [file mmc1.pdf]

Supplementary Table 1. Definition and scoring approach for the American Heart Association's Life's Essential 8 score

Domain	CVH Metric	Measurement	Quantification and Scoring of CVH Metric
Health Behaviors	Diet	Healthy Eating Index-2015 diet scores percentile	Quantiles of DASH-style diet adherence<br><br>**Scoring (Population):**<br>Points Quantile<br>100 ≥95th percentile (top/ideal diet)<br>80 75th – 94th percentile<br>50 50th – 74th percentile<br>25 25th – 49th percentile<br>0 1st – 24th percentile (bottom/least ideal quartile)
	Physical activity	Self-reported minutes of moderate or vigorous physical activity per week	**Metric:** Minutes of moderate (or greater) intensity activity per week<br><br>**Scoring:**<br>Points Minutes<br>100 ≥150<br>90 120 – 149<br>80 90 – 119<br>60 60 – 89<br>40 30 – 59<br>20 1 – 29<br>0 0
	Nicotine exposure	Self-reported use of cigarettes or inhaled nicotine- delivery system	**Metric:** Combustible tobacco use and/or inhaled NDS use; or secondhand smoke exposure<br><br>**Scoring:**<br>Points Status<br>100 Never smoker<br>75 Former smoker, quit ≥5 yrs<br>50 Former smoker, quit 1 - <5 yrs<br>25 Former smoker, quit <1 year, or currently using inhaled NDS<br>0 Current smoker<br><br>Subtract 20 points (unless scores is 0) for living with active indoor smoker in home
	Sleep health	Self-reported average hours of sleep per night	**Metric:** Average hours of sleep per night<br><br>**Scoring:**<br>Points Level<br>100 7 – <9

			90 9 – <10<br>70 6 – <7<br>40 5 – <6 or ≥10<br>20 4 – <5<br>0 <4
Health Factors	Body mass index	Body weight (kg) divided by height squared (m^2)	**Metric:** Body mass index (kg/m^2)<br><br>**Scoring:**<br><u>Points Level</u><br>100 <25<br>70 25.0 – 29.9<br>30 30.0 – 34.9<br>15 35.0 – 39.9<br>0 ≥40.0
	Blood lipids	Plasma total and HDL-cholesterol with calculation of non-HDL-cholesterol	**Metric:** Non-HDL-cholesterol (mg/dL)<br><br>**Scoring:**<br><u>Points Level</u><br>100 <130<br>60 130 – 159<br>40 160 – 189<br>20 190 – 219<br>0 ≥220<br><br>If drug-treated level, subtract 20 points
	Blood glucose	Fasting blood glucose or casual hemoglobin A1c	**Metric:** Fasting blood glucose (mg/dL) or Hemoglobin A1c (%)<br><br>**Scoring:**<br><u>Points Level</u><br>100 No history of diabetes and FBG <100 (or HbA1c < 5.7)<br>60 No diabetes and FBG 100 – 125 (or HbA1c 5.7-6.4) (Pre-diabetes)<br>40 Diabetes with HbA1c <7.0<br>30 Diabetes with HbA1c 7.0 – 7.9<br>20 Diabetes with HbA1c 8.0 – 8.9<br>10 Diabetes with Hb A1c 9.0 – 9.9<br>0 Diabetes with HbA1c ≥10.0
	Blood pressure	Appropriately measured systolic and diastolic blood pressure	**Metric:** Systolic and diastolic blood pressure (mm Hg)<br><br>**Scoring:**<br><u>Points Level</u><br>100 <120/<80 (Optimal)

			75 120-129/<80 (Elevated)<br>50 130-139 or 80-89 (Stage I HTN)<br>25 140-159 or 90-99<br>0 ≥160 or ≥100<br><br>Subtract 20 points if treated level

Reference

1. Lloyd-Jones DM, Allen NB, Anderson CAM, et al. Life's Essential 8: Updating and Enhancing the American Heart Association's Construct of Cardiovascular Health: A Presidential Advisory From the American Heart Association. Circulation. Aug 2 2022;146(5):e18-e43.
2. Lloyd-Jones DM, Ning H, Labarthe D, et al. Status of Cardiovascular Health in US Adults and Children Using the American Heart Association's New "Life's Essential 8" Metrics: Prevalence Estimates From the National Health and Nutrition Examination Survey (NHANES), 2013 Through 2018. Circulation. Sep 13 2022;146(11):822-835.

Supplementary Table 2. Healthy Eating Index-2015 Components & Scoring Standards[1]

Component	Maximum points	Standard for maximum score	Standard for minimum scores of zero
Adequacy			
**Total Fruits[2]**	5	≥0.8 cup equiv. per 1,000 kcal	No Fruit
**Whole Fruits[3]**	5	≥0.4 cup equiv. per 1,000 kcal	No Whole Fruit
**Total Vegetables[4]**	5	≥1.1 cup equiv. per 1,000 kcal	No Vegetables
**Greens and Beans[4]**	5	≥0.2 cup equiv. per 1,000 kcal	No Dark Green Vegetables or Legumes
**Whole Grains**	10	≥1.5 oz equiv. per 1,000 kcal	No Whole Grains
**Dairy[5]**	10	≥1.3 cup equiv. per 1,000 kcal	No Dairy
**Total Protein Foods[6]**	5	≥2.5 oz equiv. per 1,000 kcal	No Protein Foods
**Seafood and Plant Proteins[6,7]**	5	≥0.8 oz equiv. per 1,000 kcal	No Seafood or Plant Proteins
**Fatty Acids[8]**	10	(PUFAs + MUFAs)/SFAs ≥2.5	(PUFAs + MUFAs)/SFAs ≤1.2
Moderation			
**Refined Grains**	10	≤1.8 oz equiv. per 1,000 kcal	≥4.3 oz equiv. per 1,000 kcal
**Sodium**	10	≤1.1 gram per 1,000 kcal	≥2.0 grams per 1,000 kcal
**Added Sugars**	10	≤6.5% of energy	≥26% of energy
**Saturated Fats**	10	≤8% of energy	≥16% of energy

(1) Intakes between the minimum and maximum standards are scored proportionately.

(2) Includes 100% fruit juice.

(3) Includes all forms except juice.

(4) Includes legumes (beans and peas).

(5) Includes all milk products, such as fluid milk, yogurt, and cheese, and fortified soy beverages.

(6) Includes legumes (beans and peas).

(7) Includes seafood, nuts, seeds, soy products (other than beverages), and legumes (beans and peas).

(8) Ratio of poly- and monounsaturated fatty acids (PUFAs and MUFAs) to saturated fatty acids (SFAs).

Adequacy components represent the food groups, subgroups, and dietary elements that are encouraged. For these components, higher scores reflect higher intakes, because higher intakes are desirable.

Moderation components represent the food groups and dietary elements for which there are recommended limits to consumption. For moderation components, higher scores reflect lower intakes, because lower intakes are more desirable.

Reference

3. Krebs-Smith SM, Pannucci TE, Subar AF, et al. Update of the Healthy Eating Index: HEI-2015. J Acad Nutr Diet. Sep 2018;118(9):1591-1602.
4. National Cancer Institute. HEI Scoring Algorithm. Accessed August, 2022. https://epi.grants.cancer.gov/hei/hei-scoring-method.html

Supplementary Table 3. Collinearity analysis

Variables	Variance inflation factor (VIF)
Age	1.15
Gender	1.20
Race/ethnicity	1.17
Education	1.04
Marital status	1.10
Alcohol consumption	1.08
Energy intake	1.22
Anemia	1.05
Life’s Essential 8 score	1.09

Supplementary Table 4. Sensitivity analysis of the association between Life's Essential 8 score and diabetic retinopathy according to tertiles of Life's Essential 8 score

	Univariable model		Model 1		Model 2	
	OR (95%CI)	*P* value	OR (95%CI)	*P* value	OR (95%CI)	*P* value
Quartile 1 (0–48.75)	1[Reference]	/	1[Reference]	/	1[Reference]	/
Quartile 2 (48.76–58.13)	0.59(0.41,0.84)	0.004	0.54(0.37,0.79)	0.003	0.53(0.36,0.79)	0.004
Quartile 3 (58.14–67.50)	0.59(0.42,0.84)	0.005	0.55(0.39,0.78)	0.002	0.59(0.40,0.87)	0.009
Quartile 4 (67.51–100)	0.37(0.22,0.60)	< 0.001	0.32(0.20,0.51)	< 0.001	0.36(0.24,0.55)	< 0.001
P for trend	/	< 0.001	/	< 0.001	/	< 0.001
Per 10 points increase	0.77(0.68,0.88)	< 0.001	0.74(0.66,0.83)	< 0.001	0.77(0.69,0.84)	< 0.001

Model 1 was adjusted for age, gender, race/ethnicity, education level, and marital status;

Model 2 was additionally adjusted for alcohol consumption, energy intake, and anemia.

Abbreviations: OR, odds ratio; CI, confidence interval.

Supplementary Table 5. Survey-weighted association between Life's Essential 8 components and diabetic retinopathy

	Univariable model		Model 1		Model 2	
	**OR (95%CI)**	***P* value**	**OR (95%CI)**	***P* value**	**OR (95%CI)**	***P* value**
**Per 10 points increase**						
Diet score	1.00(0.94,1.05)	0.854	0.98(0.93,1.03)	0.475	0.99(0.94,1.04)	0.733
Physical activity score	0.97(0.93,1.00)	0.077	0.98(0.94,1.01)	0.115	0.98(0.95,1.02)	0.311
Nicotine exposure score	1.02(0.99,1.05)	0.288	1.00(0.97,1.04)	0.887	1.00(0.96,1.04)	0.862
Sleep health score	1.00(0.94,1.08)	0.887	1.01(0.94,1.08)	0.843	1.01(0.95,1.09)	0.673
Body mass index score	0.98(0.93,1.04)	0.575	0.95(0.90,1.02)	0.139	0.97(0.92,1.03)	0.330
Blood lipids score	1.07(1.00,1.14)	0.055	1.05(0.98,1.12)	0.134	1.04(0.98,1.11)	0.192
Blood glucose score	0.70(0.65,0.76)	< 0.001	0.70(0.65,0.76)	< 0.001	0.71(0.65,0.77)	< 0.001
Blood pressure score	0.90(0.85,0.94)	< 0.001	0.91(0.86,0.97)	0.004	0.91(0.86,0.97)	0.004

Model 1 was adjusted for age, gender, race/ethnicity, education level, and marital status;

Model 2 was additionally adjusted for alcohol consumption, energy intake, and anemia.

Abbreviations: OR, odds ratio; CI, confidence interval.

Supplementary Table 6. Survey-weighted association between Life's Essential 8 score and biological aging markers

	Univariable model		Model 1		Model 2	
	Beta Estimate (95%CI)	*P* value	Beta Estimate (95%CI)	*P* value	Beta Estimate (95%CI)	*P* value
**Phenotypic age**						
Low (0–49)	1[Reference]	/	1[Reference]	/	1[Reference]	/
Moderate (50–74)	-1.93(-4.52, 0.66)	0.138	-3.82(-5.26,-2.39)	< 0.001	-3.65(-5.04,-2.27)	< 0.001
High (75–100)	-7.13(-10.42,-3.84)	< 0.001	-7.30(-8.59,-6.00)	< 0.001	-6.74(-8.01,-5.47)	< 0.001
P for trend	/	< 0.001	/	< 0.001	/	< 0.001
LE8 per 10 points increase	-1.17(-1.88,-0.45)	0.002	-1.77(-2.10,-1.44)	< 0.001	-1.63(-1.93,-1.33)	< 0.001
**Biological age**						
Low (0–49)	1[Reference]	/	1[Reference]	/	1[Reference]	/
Moderate (50–74)	-11.96(-17.27, -6.66)	< 0.001	-12.35(-16.75, -7.95)	< 0.001	-11.95(-16.13, -7.77)	< 0.001
High (75–100)	-25.77(-32.44,-19.10)	< 0.001	-24.38(-29.84,-18.92)	< 0.001	-23.10(-28.67,-17.52)	< 0.001
P for trend	/	< 0.001	/	< 0.001	/	< 0.001
LE8 per 10 points increase	-6.23(-7.52,-4.94)	< 0.001	-6.40(-7.36,-5.43)	< 0.001	-6.10(-7.08,-5.12)	< 0.001

Model 1 was adjusted for age, gender, race/ethnicity, education level, and marital status;

Model 2 was additionally adjusted for alcohol consumption, energy intake, and anemia.

Abbreviations: CI, confidence interval.

Supplementary Table 7. Survey-weighted association between biological aging markers and diabetic retinopathy

	Univariable model		Model 1		Model 2	
	OR (95%CI)	*P* value	OR (95%CI)	*P* value	OR (95%CI)	*P* value
**Phenotypic age**						
Tertile 1 (< 53.09)	1[Reference]	/	1[Reference]	/	1[Reference]	/
Tertile 2 (53.10–65.52)	2.90(1.81,4.65)	< 0.001	4.63(2.72, 7.90)	< 0.001	4.14(2.31,7.43)	< 0.001
Tertile 3 (≥ 65.53)	4.43(2.85,6.88)	< 0.001	11.34(4.43,29.04)	< 0.001	8.73(3.41,22.35)	< 0.001
P for trend	/	< 0.001	/	< 0.001	/	< 0.001
Per 1 year increase	1.04(1.03,1.06)	< 0.001	1.08(1.05,1.11)	< 0.001	1.07(1.05,1.10)	< 0.001
**Biological age**						
Tertile 1 (< 38.35)	1[Reference]	/	1[Reference]	/	1[Reference]	/
Tertile 2 (38.36–56.54)	2.01(1.19,3.38)	0.011	1.85(1.08,3.18)	0.027	1.77(1.01,3.10)	0.046
Tertile 3 (≥ 56.55)	4.08(2.73,6.12)	< 0.001	3.38(2.26,5.05)	< 0.001	2.99(2.04,4.39)	< 0.001
P for trend	/	< 0.001	/	< 0.001	/	< 0.001
Per 1 year increase	1.03(1.02,1.03)	< 0.001	1.02(1.02,1.03)	< 0.001	1.02(1.01,1.03)	< 0.001

Model 1 was adjusted for age, gender, race/ethnicity, education level, and marital status;

Model 2 was additionally adjusted for alcohol consumption, energy intake, and anemia.

Abbreviations: OR, odds ratio; CI, confidence interval.

Supplementary Table 8. The mediating proportion of biological aging markers on the association between Life's Essential 8 score and diabetic retinopathy

**Model Pathways**	Mediating Effect		
	Beta Estimate (95%CI)	*P* value	Proportion Mediated (%)
**Phenotypic age**			
Total effect	-0.0061(-0.0077,-0.0038)	< 0.001	100
Direct effect	-0.0039(-0.0059,-0.0017)	< 0.001	64.39
Indirect effect	-0.0022 (-0.0029,-0.0014)	< 0.001	35.61
**Biological age**			
Total effect	-0.0060(-0.0076,-0.0037)	< 0.001	100
Direct effect	-0.0032(-0.0054,-0.0008)	0.014	53.62
Indirect effect	-0.0028(-0.0036,-0.0018)	< 0.001	46.38

Adjusted for age, gender, race/ethnicity, education level, marital status, alcohol consumption, energy intake, and anemia. Abbreviations: *CI*, confidence interval.

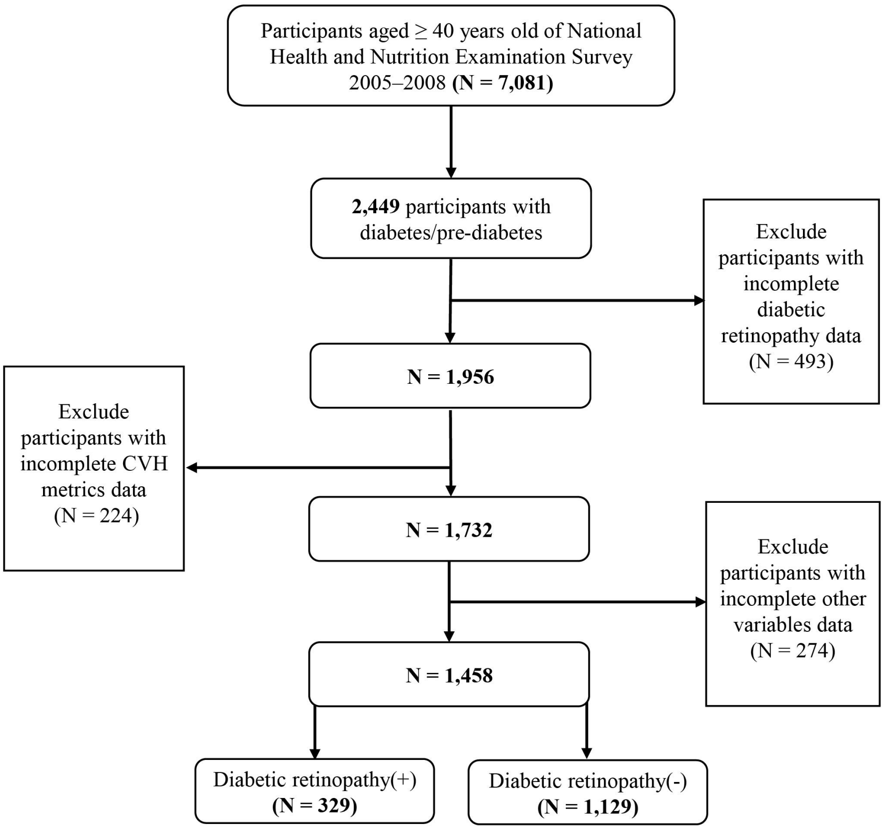

Supplementary Figure 1. Study flowchart.

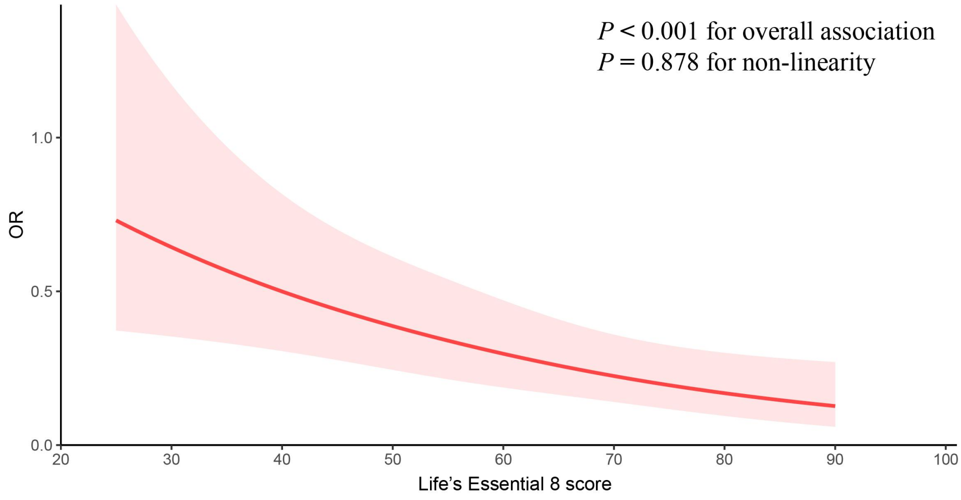

Supplementary Figure 2. Dose-response relationships between Life's Essential 8 score and diabetic retinopathy.

Adjusted for age, gender, race/ethnicity, education level, marital status, alcohol consumption, energy intake, and anemia. The shaded part represents the 95% CI.

Abbreviations: OR, odds ratio; CI, confidence interval.

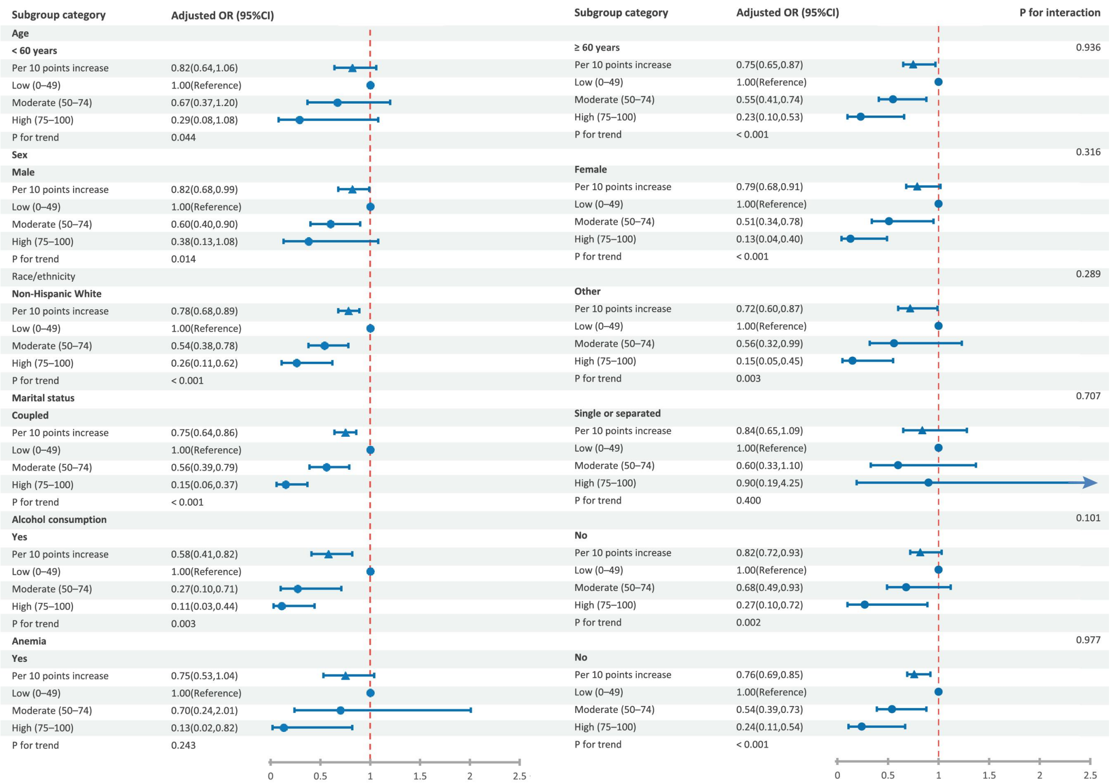

Supplementary Figure 3. Subgroup analyses of the association of Life's Essential 8 score with diabetic retinopathy.

Adjusted for age, gender, race/ethnicity, education level, marital status, alcohol consumption, energy intake, and anemia. Abbreviations: OR, odds ratio; CI, confidence interval.
